# Unique Author Identification Number in Scientific Databases: A Suggestion

**DOI:** 10.1371/journal.pmed.0030249

**Published:** 2006-05-30

**Authors:** Matthew E Falagas

**Affiliations:** **1**Alfa Institute of Biomedical Sciences (AIBS)AthensGreece

There is an increasing trend toward the use of electronic databases of scientific information, such as the PubMed database of the National Library of Medicine and the various databases of the Institute for Scientific Information (ISI). These databases are frequently used nowadays for various purposes, including the peer-review process of papers submitted for publication in scientific journals. Most of the scientific journals now use a Web-based peer-review system that offers editors, peer reviewers, and publishers the capability to check the previous papers published by authors submitting a manuscript for consideration for publication [ 1]. In addition, these databases are frequently searched in an attempt to select potential speakers for scientific conferences and to obtain data for possible collaborators for a multicenter study, as well as in the process of evaluating the research productivity of scientists—which is being used for various purposes.

However, it is widely known that a considerable proportion of authors share the same last name and first initial. This seems to be the case for people of most ethnic heritages. In addition, authors of scientific publications do not frequently use their middle initial, which contributes to the confusion regarding the assignment of publications to the appropriate author. Frequently, it takes considerable effort and time to assign publications to the appropriate authors, particularly if there are authors that share the same last name and first initial (with or without the middle initial). This is usually based on the pattern of research interests, as well as the institutional affiliations of the various authors with the same name. In fact, this task is often impossible.

In order to decrease the problems arising from authors with identical names, I suggest the introduction of a unique author identification number (UAIN) in modern electronic databases of scientific information. I further suggest that such an identification number may be hidden in the electronic databases, i.e., it is not necessary for the UAIN to appear when reviewing the record of a publication. This function of the electronic databases could start operating after the providers of the databases are given a reasonable time to prepare. To avoid the resources needed to update the electronic databases with the appropriate assignment of publications to each author, I suggest that the UAIN not be used retrospectively. In this case, the record of publications for a specific author would be divided into two parts. The publications that are prospectively connected to a given author with a specific UAIN would have an indicator denoting this, while older publications (before the introduction of the UAIN) would not have such an indicator.
